# P-1927. Utilization of Patient Characteristics and the Area Deprivation Index (ADI) To Evaluate Referral Patterns for Long COVID at a Tertiary-Care Veterans Affairs (VA) Medical Center

**DOI:** 10.1093/ofid/ofae631.2087

**Published:** 2025-01-29

**Authors:** Martha Brennan, Brigid Wilson, Usha Stiefel

**Affiliations:** VA Northeast Ohio Healthcare System, Cleveland, Ohio; VA Northeast Ohio Healthcare System, Cleveland, Ohio; VA Northeast Ohio Healthcare System, Cleveland, Ohio

## Abstract

**Background:**

The COVID-19 pandemic disproportionally affected socioeconomically disadvantaged communities, with corollary expectation of increased impact of post-acute sequelae in this population. We established a subspecialty Long COVID Clinic (LCC) at VA Northeast Ohio to care for patients with post-acute sequelae of COVID-19 in 2023. The LCC was advertised system-wide to providers via email and videoconference presentations, and to patients via the facility website. After the first 1/2 year of implementation, an analysis of patients and of referral adequacy in meeting the needs of the catchment area was undertaken.

**Figure:**
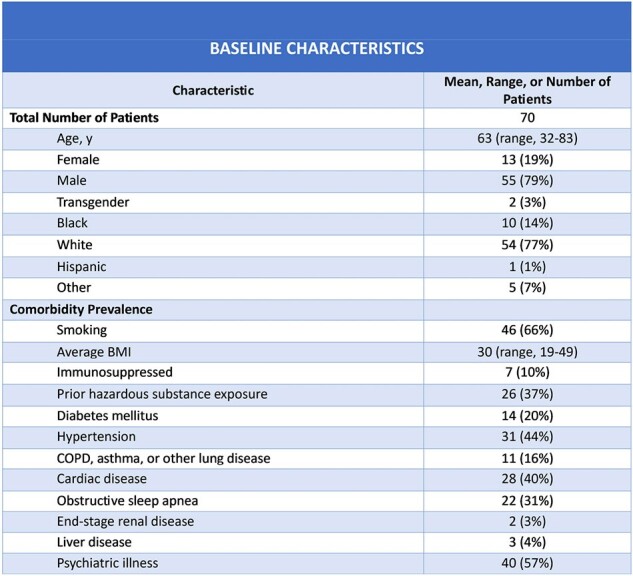

**Methods:**

The Area Deprivation Index (ADI) uses census data to rank US neighborhoods by socioeconomic disadvantage and can be a tool in refining delivery of services or healthcare. Neighborhoods are ranked nationally and within-state (state deciles 1-10, with 10 indicating greatest disadvantage). In addition to other health factors, national and state ADI indices were obtained for each patient seen in our LCC between March 2023 and April 2024.

**Figure:**
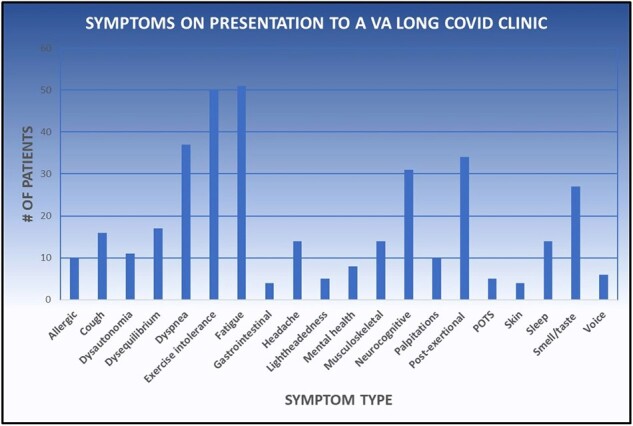

**Results:**

70 unique patients (mean age 63) were seen via referral in the 1^st^ 8 months of the LCC (Fig 1). These were 13 (19%) women, 55 (79%) men, 2 (1%) transgender, 10 (14%) Black and 54 (77%) White. The most common comorbidities were psychiatric (57%), hypertension (44%) and cardiac (40%) (Fig 1). The most common presenting complaints were fatigue (73%), exercise intolerance (72%), and dyspnea (52%) (Fig 2). ∼1/4 of LCC patients had experienced severe COVID-19 (Fig 3). The average in-state ADI decile was 5 (range, 1-10).

**Figure:**
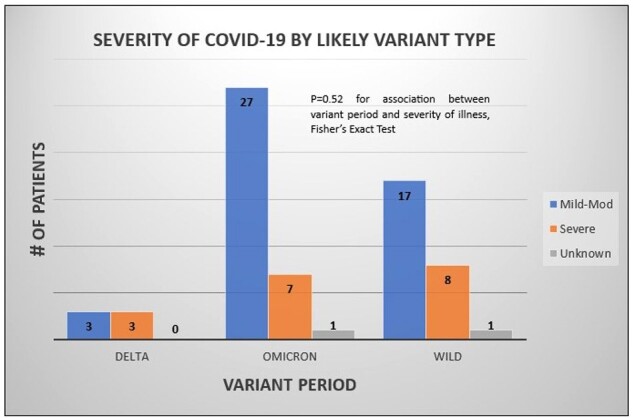

**Conclusion:**

1. Women Veterans, who make up 9% of the Veteran population, were overrepresented in an early referral population to our Long COVID Clinic.
2. Certain populations, including women and patients with psychiatric illness, stand out as candidates for enhanced screening for symptoms after COVID-19.
3. The average within-state ADI decile of patients in our LCC was right at the 50^th^ percentile, suggesting that health-marginalized populations may not be accessing care for post-acute sequelae of COVID-19 at the appropriate frequency, considering the expected burden of disease.
4. The ADI, along with other health factors, can identify opportunities to improve penetration of clinical Long COVID care across a VA catchment area.

**Disclosures:**

All Authors: No reported disclosures

